# Response: Reply to the commentary on our study about respiratory muscle strength in stroke patients

**DOI:** 10.1590/1806-9282.20250920

**Published:** 2025-10-17

**Authors:** Abdurrahim Yildiz, Rustem Mustafaoglu, Ayşe Nur Bardak

**Affiliations:** 1 Sakarya University of Applied Sciences, Department of Physiotherapy and Rehabilitation – Sakarya, Türkiye.; 2 Istanbul University-Cerrahpaşa, Faculty of Health Sciences, Department of Physiotherapy and Rehabilitation – Istanbul, Türkiye.; 3 University of Health Sciences, Istanbul Physical Medicine and Rehabilitation Training and Research Hospital – Istanbul, Türkiye.

Dear Editor,

We thank Dr. Josef Finsterer for his valuable comments^1^. We are pleased that our study has generated scientific discussion. Below, we provide our detailed responses to the points raised.

We acknowledge that respiratory muscle weakness may result from a wide range of underlying conditions, and that these differential diagnoses must be carefully excluded before attributing the weakness to chronic stroke. In light of this, our study was designed with strict exclusion criteria to minimize potential confounders. Specifically, patients with a diagnosis of any pulmonary disease, cardiovascular disease, or other neurological disorders were excluded to ensure that observed respiratory muscle impairments could be more confidently associated with chronic stroke. Additionally, numerous factors can influence respiratory muscle strength. According to the literature, these include variables such as age, body mass index^2-4^, impaired motor function, reduced thoracic expansion, postural trunk dysfunction, and decreased lung volumes^5^, as well as muscle atrophy due to physical inactivity^6,7^, spasticity, and impaired trunk stability^8,9^. Given the multifactorial nature of respiratory muscle weakness, it is practically impossible to design a study that can completely eliminate the influence of all these variables.

We partially agree with this perspective, as stroke localization may influence pulmonary function. While some studies in the literature support this association, others emphasize that the relationship is complex and multifactorial. Moreover, many studies do not report stroke localization in detail. In the present study, no subgroup analysis was conducted based on stroke localization. However, future research may benefit from more specific subgrouping according to lesion site, which could help elucidate the characteristic respiratory profiles associated with different stroke localizations.

The mean duration (388 days) is presented, and the range and standard deviation are available in our original data. The omission of this information in the published manuscript may be considered a limitation. However, the inclusion of patients specifically in the chronic phase of stroke, where the clinical condition is generally stable, was intended to minimize the likelihood that factors other than stroke would account for reduced maximal inspiratory pressure (MIP) or maximal expiratory pressure (MEP). Therefore, we believe that reporting how many of the participants experienced intervening illnesses after stroke that could potentially affect MIP or MEP would not provide substantial additional value to our study. It is not feasible to conduct a detailed investigation into how many new illnesses each participant developed, what exposures they encountered, or what events they experienced following their stroke. Even if such information were obtainable, it would be impractical to include that level of detail in a scientific manuscript. While every effort is made to control for potential confounders, it is not possible to account for every variable in clinical research involving human participants.

In our study, we aimed to achieve approximate matching between the stroke and control groups based on age and gender distributions, rather than performing strict one-to-one matching or maintaining equal group sizes. This approach is commonly used in observational research^10,11^, particularly when the case group is considerably larger and the control group is limited due to recruitment challenges. Regarding the noted difference in gender ratios, we acknowledge that there is a discrepancy: the proportion of males was 39% in the stroke group and 56% in the control group. While we initially considered this difference to be within an acceptable range for observational research, we recognize that this variation may introduce potential confounding. We therefore accept this as a limitation of our study. In future research, we intend to apply more stringent matching or statistical adjustment techniques (e.g., propensity score matching or multivariable regression) to better control for such demographic imbalances. On the other hand, in the methodological analyses conducted by Edwardes, it was emphasized that keeping the number of cases high contributed significantly to the statistical power, whereas the increase in the number of controls made a limited contribution. In this context, the small number of the control group does not seriously affect the reliability of the results, especially when the case group is large enough^12^. In addition, as can be seen from the tables of our study, male stroke patients were compared with male healthy subjects and female stroke patients were compared with female healthy subjects.

Given that this study is retrospective in nature and covers the period between 2017 and 2021 — including the COVID-19 pandemic — patients with documented SARS-CoV-2 infection or a history of mechanical ventilation during the study period were evaluated under the category of pulmonary disease and were therefore excluded from the study.

In the study, the term chronic stroke refers to individuals who had experienced a stroke at least 6 months prior to enrollment and were in the chronic recovery phase, characterized by stabilized neurological deficits. This definition is consistent with the commonly accepted classification in stroke rehabilitation literature. We included only patients with a confirmed diagnosis of ischemic stroke, based on clinical and radiological findings. Patients with a history of cerebral hemorrhage (hemorrhagic stroke) were excluded from the study to maintain sample homogeneity and reduce confounding variables related to different pathophysiological mechanisms and recovery profiles.

We sincerely appreciate your valuable comments, which contribute to presenting our study more clearly and guiding future research directions. We also thank you for your constructive scientific input. However, it is important to acknowledge that every study has its limitations, and no research can be entirely free of constraints. Within the scope of our professional and academic expertise — and under the given conditions — we have endeavored to conduct this study to the highest standards possible. We remain open to collaboration and dialog to further improve the quality and impact of future research in this field.

## Data Availability

The datasets generated and/or analyzed during the current study are available from the corresponding author upon reasonable request.
